# Early Cretaceous *Archaeamphora* is not a carnivorous angiosperm

**DOI:** 10.3389/fpls.2015.00326

**Published:** 2015-05-07

**Authors:** William Oki Wong, David Leonard Dilcher, Conrad C. Labandeira, Ge Sun, Andreas Fleischmann

**Affiliations:** ^1^State Key Laboratory of Systematic and Evolutionary Botany, Institute of Botany, Chinese Academy of Sciences, Beijing, China; ^2^Department of Geology, Indiana University, Bloomington, IN, USA; ^3^Department of Paleobiology, National Museum of Natural History, Washington, DC, USA; ^4^Department of Entomology and BEES Program, University of Maryland, College Park, MD, USA; ^5^College of Life Sciences, Capital Normal University, Beijing, China; ^6^Paleontological Institute, Shenyang Normal University, Shenyang, China; ^7^Botanische Staatssammlung München, Munich, Germany

**Keywords:** angiosperm, *Archaeamphora longicervia*, carnivorous plant, Early Cretaceous, leaf gall, *Liaoningocladus boii*, Sarraceniaceae, Yixian Formation

## Abstract

*Archaeamphora longicervia* H. Q. Li was described as an herbaceous, Sarraceniaceae-like pitcher plant from the mid Early Cretaceous Yixian Formation of Liaoning Province, northeastern China. Here, a re-investigation of *A. longicervia* specimens from the Yixian Formation provides new insights into its identity and the morphology of pitcher plants claimed by Li. We demonstrate that putative pitchers of *Archaeamphora* are insect-induced leaf galls that consist of three components: (1) an innermost larval chamber; (2) an intermediate zone of nutritive tissue; and (3) an outermost wall of sclerenchyma. *Archaeamphora* is not a carnivorous, Sarraceniaceae-like angiosperm, but represents insect-galled leaves of the previously reported gymnosperm *Liaoningocladus boii* G. Sun et al. from the Yixian Formation.

## Introduction

Li (2005) described *Archaeamphora longicervia* H. Q. Li as an “Early Cretaceous sarraceniacean-like pitcher plant” from the Yixian Formation of Liaoning Province in northeastern China. These fossils were interpreted by Li as an herbaceous plant having spirally arranged, ascidiate pitchers and phyllodia-like leaves with parallel veins, similar to modern Sarraceniaceae, a New World carnivorous plant family of Ericales represented by three extant genera: *Sarracenia* L., *Darlingtonia* Torr., and *Heliamphora* Benth. (Lloyd, 1942; Juniper et al., 1989; Schnell, 2002). However, doubts have been cast on whether *Archaeamphora* actually represents an early sarraceniacean plant, because its age contradicts the divergence time estimate, phylogeny, and biogeography of carnivorous Ericalean lineages (Fleischmann, 2010; Ellison et al., 2012; Brittnacher, 2013; Givnish, 2015; Sadowski et al., 2015). Here, we propose a new perspective on the morphology, identity, and affinity of *A. longicervia* in light of a re-investigation of newly collected Yixian specimens.

## New Fossils of *Archaeamphora longicervia*

**FIGURE 1:**
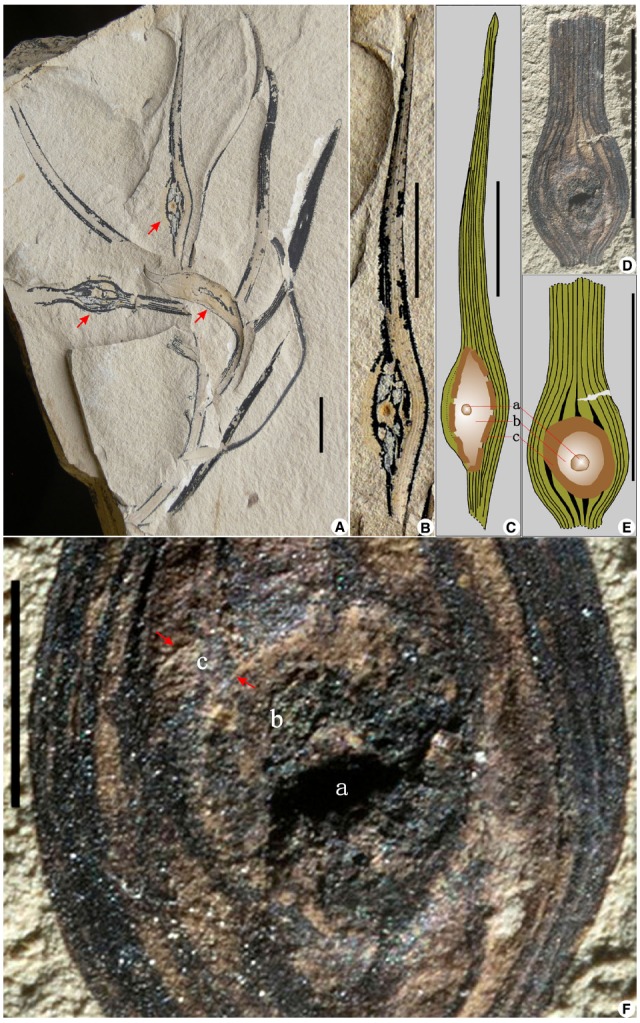
**A leaf gall from the Early Cretaceous Yixian Formation of Liaoning, northeastern China. (A)** A distal shoot with normal and galled leaves. Red arrows refer to three galls. **(B)** Partial enlargement of an upper gall in **(A)**. **(C)** An overlay drawing of the gall in **(B)**, showing gall features in cross-section. **(D)** A detached leaf fragment with a gall, showing tufts of nutritive tissue surrounding the innermost chamber and encapsulated by the outer sclerenchyma. **(E)** An overlay drawing of the gall in **(D)**, with same features as in **(C)**. **(F)** Partial enlargement of the gall in **(D)**. Red arrows refer to the outermost sclerenchymatous layer of the gall. **(a)** Innermost larval chamber; **(b)** intermediate zone of gall nutritive tissue; **(c)** outermost sclerenchymatous layer. Scale bars = 1 cm, except in **(F)**, which is 2 mm.

The two fossils presented here (Figure 1) were collected from the Jianshangou Bed of the Yixian Formation at Huangbanjigou Village near Beipiao City, Liaoning Province, northeastern China (Sun et al., 1998, 2001, 2011; Yang and Wang, 2013). The geological age of the Jianshangou Bed within the Yixian Formation can be established by multiple radiometric dates to ca. 122–125 Ma (Swisher et al., 2002; Zhou et al., 2003; Chang et al., 2009) or 125–127 Ma (Sun et al., 2011), which correlates to the Early Cretaceous late Barremian–early Aptian Stages. The fossils are preserved as compressions/impressions, with minimal carbonized organic material remaining. They were photographed with Nikon D700 and Panasonic DMC-FZ30 digital cameras, and fossil overlay drawings were drawn using the software Corel-Draw 10.0 (Corel Corporation, Ottawa, ON, Canada). Voucher specimens are deposited in the Indiana University Paleontology Type Collection, Department of Geology, Indiana University, Bloomington, IN, USA.

## A New Perspective on *Archaeamphora longicervia*

The two fossils represent a distal shoot bearing spirally arranged leaves and a detached leaf fragment, respectively (Figures 1A,D). These leaves are lanceolate in form, about 50–80 × 2–6 mm in size, with nearly parallel veins, ca. 4–11 in number at the widest portions of each leaf, usually forking once near slightly constricted leaf bases, but unbranched and converging toward the acute or blunt apexes. The parallel veins on different parts of four leaves have been interrupted by a swollen, bulbous structure (ca. 4–10 × 4–5 mm in size), each of which is usually bordered by a grossly elliptical to orbicular ring zone, which often encloses a tiny, spheroidal lump or depression, about 0.8 mm in diameter (Figures 1B–F). Li (2005) interpreted such structures as pitchers of the carnivorous plant *Archaeamphora longicervia*. Instead, we explain these structures as foliar galls, which are abnormal swellings of plant tissues that contrast dramatically with normally developed leaves present on the shoot of the same specimens (Figure 1A). The plant host morphologically is more or less consistent with the Yixian conifer *Liaoningocladus boii* G. Sun et al., which bears helically arranged, narrowly lanceolate leaves (ca. 20–70 mm long by 2–5 mm wide) with a tapering to blunt apex, a somewhat contracted base, and about 4–11 in number of nearly parallel veins (Sun et al., 2000, 2001). These swollen, hemispheroidal to hemi-ellipsoidal leaf galls typically include an innermost larval chamber (Figures 1Ca,Ea,Fa) that would house the gall-inducing insect. A second, intermediately positioned zone (Figures 1Cb,Eb,Fb) is the parenchymatous nutritive tissue that is consumed by the gall-inducing insect and houses tissue with proliferative files of hyperplasic and hypertrophic cells (Labandeira and Phillips, 2002). A third major gall zone (Figures 1Cc,Ec,Fc) is the protective, hardened, sclerenchyma forming the outermost layer (Labandeira and Phillips, 2002).

## Discussion

The age and place of origin of *Archaeamphora* are inconsistent with data retrieved from phylogenetic reconstructions of Sarraceniaceae and Ericales, respectively. Molecular clock dating, based on robust phylogenetic data, estimates an Eocene divergence time of 44–53 Ma for the Sarraceniaceae lineage (Ellison et al., 2012). This age estimate is further corroborated by fossil evidence from the sister group of Sarraceniaceae, Roridulaceae, that occur as leaves from Eocene Baltic amber dated at 35–47 Ma (Sadowski et al., 2015). Therefore, 122–127 million-year-old Cretaceous *Archaeamphora* is inordinately too old to be a member of the phylogenetically much younger Sarraceniaceae, or even Ericales. The Ericales is a lineage of core crown eudicots estimated at 92–102 Ma (Bell et al., 2010). By contrast, basal eudicots are considered to extend to the Barremian–Aptian boundary, corresponding to an age of 125 Ma (Sun et al., 2011; Wang et al., 2015). Also, the Clethraceae–Sarraceniaceae–Roridulaceae–Actinidiaceae clade is very likely to have originated in America (Ellison et al., 2012), rendering unlikely the assignment of Early Cretaceous *Archaeamphora* to the Sarraceniaceae of China.

All pitcher leaves of carnivorous plants are based upon epiascidiate leaves during ontogeny (Arber, 1941; Lloyd, 1942; Franck, 1975; Juniper et al., 1989; Fukushima et al., 2015) By contrast, the hollow, spheroidal to ellipsoidal cavities of *Archaeamphora* result from insect-induced galling. The “alae” of the pitcher, suggested by Li (2005), simply represent the foliar part of the uninfected gall. Li’s (2005) interpretation of different trapping “zones,” present in extant Sarraceniaceae pitchers, is untenable for the fossil leaves. A “fluid filled zone” at the bottom of the leaf interior, as suggested by Li (2005), is extremely unlikely due to the very narrow diameter (3–5 mm) of the cavity, where a constant water level would not be maintained due to capillary forces. The pitcher morphology of extant Sarraceniaceae is relatively constant at the species level. Li (2005) reported different “types of pitchers,” which he inferred to constitute different taxa that diversified before the Early Cretaceous. Instead, the variability in “pitcher” structure supports our explanation that these features result from more variable, tumor-like insect-induced growth reactions of the leaves, particularly the proliferation of nutritive tissue (Meyer, 1987). Because the position of the gall lacks constancy along the length of the leaf, the size of the “neck” part, as suggested by Li (2005), is similarly variable.

Insect-induced gall fossils provide unique opportunities for studying ecological links between first and second trophic levels in food chains, and the third trophic level of galls with parasitoid exit holes (Labandeira, 2002). The galling behavior of arthropods on plants has occurred in two mite lineages (Eriophyidae, Tetranychidae), and in the six major insect lineages Thysanoptera, Hemiptera, Coleoptera, Diptera, Lepidoptera, and Hymenoptera (Meyer, 1987; Shorthouse and Rohfritsch, 1992; Labandeira, 1998, 2002). The attacking insect is not only highly specific in selecting a particular host species, but often selects a certain host organ or tissue, and is associated with a distinctive structure, color, and physiology of the gall (Meyer, 1987; Shorthouse and Rohfritsch, 1992; Raman, 2011). The leaf galls presented here have been discovered from the host plant *Liaoningocladus boii*, which also hosts a beetle leaf miner (Ding et al., 2014), representing only two of the numerous associations occurring on this plant host elsewhere from the Yixian Formation (Sun et al., 2001).

## Conclusion

Early Cretaceous *Archaeamphora longicervia* is not a carnivorous, Sarraceniaceae-like angiosperm, but represents insect-galled leaves of the fossil gymnosperm *Liaoningocladus boii* from the Yixian Formation of northeastern China.

## Author Contributions

WW and DD designed the research plan. WW, CL, and AF wrote the manuscript. WW and DD photographed specimens. CL identified the gall and its tissues. WW drew line-drawings and arranged the figure. All authors performed analyses, commented the text and approved the final manuscript.

### Conflict of Interest Statement

The reviewer Chunlin Sun declares that, despite having collaborated with the author David Leonard Dilcher, the review process was handled objectively. The authors declare that the research was conducted in the absence of any commercial or financial relationships that could be construed as a potential conflict of interest.
